# Mirtazapine Treatment of a Severe Depressive Episode and Resolution of Elevated Inflammatory Markers

**DOI:** 10.1155/2013/697872

**Published:** 2013-10-09

**Authors:** Shahzad M. Alikhan, Jessica A. Lee, Luiz Dratcu

**Affiliations:** ^1^ST4 in General Adult Psychiatry, Bracton Centre, Oxleas NHS Foundation Trust, Dartford, Kent DA2 7AF, UK; ^2^South West London and St. George's Mental Health NHS Trust, Springfield University Hospital, 61 Glenburnie Road Tooting, London SW17 7DJ, UK; ^3^South London and Maudsley NHS Foundation Trust, Maudsley Hospital, Denmark Hill, London SE5 8AZ, UK

## Abstract

Depression has been shown to be associated with systemic inflammatory activity and the mode of action of several antidepressants appears to involve immunomodulation. Effects on immune system activity have also recently been observed in correlation with therapeutic response to mirtazapine in cardiac patients with depression, but no study has yet examined these effects in otherwise physically healthy depressed patients treated with mirtazapine. This report describes an association between a clinical antidepressant response and a decrease in markers of systemic inflammation observed during pharmacotherapy with mirtazapine in a severely depressed but physically well patient. This observation adds to the evidence that changes in inflammatory responses may be implicated in the mode of action of antidepressants. Further studies of antidepressant responses to mirtazapine and levels of inflammatory markers in depressed patients without medical comorbidity can help elucidate the role of the immune system in the pathophysiology of depression, and hence contribute to the development of novel antidepressant therapies.

## 1. Introduction

Major depressive disorder (MDD) is a complex illness associated with immune system involvement and a systemic inflammatory response [1], including elevated C-reactive protein (CRP) and plasma immunomodulatory cytokine levels [2, 3]. It has been demonstrated that neurocytokines can induce “sickness behavior,” which mirrors the core biological and behavioural symptoms of clinical depression [4]. There is growing evidence that not only tricyclic antidepressants (TCAs) and selective serotonin reuptake inhibitors (SSRIs) but also newer noradrenergic and specific serotonergic antidepressants (NaSSAs) such as mirtazapine may modulate the inflammatory response as part of their mode of action [5]. Reduction in the levels of inflammatory markers such as CRP, the Erythrocyte Sedimentation Rate (ESR), and White Cell Count (WCC) has been observed in direct response to SSRI treatment, although it remains unclear how this relates to the therapeutic effect of antidepressants [6].

A recent study of mirtazapine treatment of postmyocardial infarction depression found a strong association between antidepressant response to mirtazapine and its effects on systemic inflammation as measured by soluble Tumour Necrosis Factor Receptor-1 (sTNF-R1) levels [7]. However, little is known about inflammatory responses to mirtazapine in depressed patients without physical comorbidity. We describe a patient with a severe depressive episode and no history or evidence of physical illness, whose successful antidepressant response to mirtazapine was accompanied by the resolution of elevated peripheral inflammatory markers.

## 2. Case Report

A 51-year-old Caucasian man was admitted to hospital due to concerns about his increasing isolation and self-neglect. He described a three month history of feeling depressed, with diurnal variation in mood, sleep disturbance, early morning wakening, anergia, anhedonia, anorexia, poor concentration, and frequent suicidal thoughts. He admitted to having superficially self-harmed and taken an overdose of 14 tablets of paracetamol 500 mg two weeks prior to admission. Full assessment of his clinical history and mental state indicated that he met ICD-10 criteria for a severe depressive episode without psychotic symptoms. He had no past medical or psychiatric history of note other than a mild depressive episode six years ago, treated briefly with paroxetine. He took no regular medications and was a nonsmoker with no history of alcohol or illicit substance misuse. In his family history, his mother had been diagnosed with bipolar affective disorder.

On day 1 of his admission, he presented as severely depressed, with self-neglect and psychomotor retardation. He reported low mood, suicidal thoughts, and was socially withdrawn with poor eye contact. Blood tests demonstrated significantly elevated inflammatory markers; specifically, acute phase proteins C-Reactive Protein (CRP) 93.6 mg/L (reference range 0–5 mg/L), serum ferritin 809 mcg/L (reference range 20–300 mcg/L), and the Erythrocyte Sedimentation Rate (ESR) 39 mm/hr (generally accepted upper limit for men = Age/2 = 25 mm/hr). 

Results of all other laboratory investigations were within normal limits, including full blood count, thyroid, renal and liver function tests, hepatitis B and C, treponemal and Human Immunodeficiency Virus (HIV) serology, autoantibody screen and Anti-Neutrophil Cytoplasmic Antibodies (ANCA), as were his electrocardiogram and urinalysis. On serial assessments, the patient denied any symptoms of infection, generalised pain, or inflammation, with repeated comprehensive physical examinations revealing no signs of such.

Treatment with mirtazapine 15 mg daily was commenced, increased on day 5 to 30 mg. By day 9, he displayed noticeable improvement of his mood, appetite, motivation, energy levels, and sleep. Blood tests at this stage showed a decrease in the CRP to 38.3 mg/L (ESR and ferritin were not requested). By day 18, mirtazapine was increased to a 45 mg daily dose. At this stage his depressive symptoms had entirely abated and the patient was considered in remission. The values of inflammatory indices were also markedly reduced: ESR to 16 mm/hr, ferritin to 428 mcg/L, and, most remarkably, a decrease of his CRP to <5 mg/L. Mirtazapine remained the sole pharmacological treatment that he had received; no anti-inflammatory, antimicrobial/antiviral, or any other medication was prescribed at any stage.

## 3. Discussion

The role of the immune system in major depression is undergoing scrutiny, having evolved to include a “cytokine theory” of depression and a characterisation of the illness as a psychoneuroimmunological disorder [8]. The anti-inflammatory properties of SSRIs have been documented [6, 9, 10], although the extent to which these correlate with the effects of these drugs on depressive symptoms remains uncertain. Response to antidepressant treatment using mirtazapine, however, has been shown to correlate with significant effects on inflammatory cytokines in a double-blind placebo controlled study of patients with depression postmyocardial infarction [7]. Although the study found no difference between experimental and control groups in circulating CRP levels, a significant increase in sTNF-R1 was observed in mirtazapine responders, thereby suggesting that the antidepressant effect of mirtazapine could be mediated by sTNF-R1 and associated immune responses. The lack of difference in CRP levels in that study could be explained by the fact that elevated CRP levels have been identified as an independent risk factor for cardiovascular disease [11] and implicated in cardiovascular outcomes.

This report, on the other hand, describes the normalisation of elevated peripheral inflammatory markers, including CRP, associated temporally with good antidepressant response to mirtazapine in a severely depressed patient with no detectable physical comorbidity and no cardiovascular problems. The association of ESR, shown to correlate positively with inflammatory cytokine levels [12], and CRP normalisation with good antidepressant response to mirtazapine seems to lend support to the view [7] that mirtazapine's antidepressant effect may be related to its anti-inflammatory properties [13]. 

Mirtazapine and other antidepressants are thought to exert their therapeutic effect by acting not only on central monoamines, particularly serotonin and noradrenaline but also on neurotrophins, like brain-derived neurotrophic factor (BDNF) [14]. This report adds to the growing evidence that changes in inflammatory responses may also be implicated in the mode of action of antidepressants. Further studies on the association of antidepressant responses with mirtazapine and changes in inflammatory markers in depressed patients without medical comorbidity can help elucidate further the role of the immune system in the pathophysiology of depression, and hence contribute to the development of new antidepressant treatments. 
